# Impact of cytomegalovirus reactivation on relapse and survival in patients with acute leukemia who received allogeneic hematopoietic stem cell transplantation in first remission

**DOI:** 10.18632/oncotarget.7347

**Published:** 2016-02-12

**Authors:** Jae-Ho Yoon, Seok Lee, Hee-Je Kim, Young-Woo Jeon, Sung-Eun Lee, Byung-Sik Cho, Dong-Gun Lee, Ki-Seong Eom, Yoo-Jin Kim, Chang-Ki Min, Seok-Goo Cho, Woo-Sung Min, Jong Wook Lee

**Affiliations:** ^1^ Department of Hematology, Catholic Blood and Marrow Transplantation Center, Cancer Research Institute, College of Medicine, The Catholic University of Korea, Seoul, Korea; ^2^ Division of Infectious Diseases, Department of Internal Medicine, Seoul St. Mary's Hospital, College of Medicine, The Catholic University of Korea, Seoul, Korea

**Keywords:** acute myeloid leukemia, acute lymphoid leukemia, cytomegalovirus, graft-vs-leukemia

## Abstract

Cytomegalovirus (CMV)-reactivation is associated with graft-vs-leukemia (GVL) effect by stimulating natural-killer or T-cells, which showed leukemia relapse prevention after hematopoietic stem cell transplantation (HSCT). We enrolled patients with acute myeloid leukemia (*n* = 197) and acute lymphoid leukemia (*n* = 192) who underwent allogeneic-HSCT in first remission. We measured RQ-PCR weekly to detect CMV-reactivation and preemptively used ganciclovir (GCV) when the titer increased twice consecutively, but GCV was sometimes delayed in patients without significant graft-vs-host disease (GVHD) by reducing immunosuppressive agents. In the entire group, CMV-reactivation showed poor overall survival (OS). To evaluate subsequent effects of CMV-reactivation, we excluded early relapse and deaths within 100 days, during which most of the CMV-reactivation occurred. Untreated CMV-reactivated group (*n* = 173) showed superior OS (83.8% vs. 61.7% vs. 74.0%, *p* < 0.001) with lower relapse rate (10.1% vs 22.1% vs. 25.5%, *p* = 0.004) compared to GCV-treated CMV-reactivated group (*n* = 122) and CMV-undetected group (*n* = 42). After excluding chronic GVHD, untreated CMV-reactivated group still showed lower relapse rate (9.4% vs. 24.1% vs. 30.2%, *p* = 0.006). Multivariate analysis showed adverse-risk karyotype and patients in other than untreated CMV-reactivated group were independent factors for relapse prediction. Our data showed possible GVL effect of CMV-reactivation and minimizing antiviral therapy may benefit for relapse prevention in acute leukemia.

## INTRODUCTION

There were reports which showed cytomegalovirus (CMV) reactivation may have a role in reduction of relapse in acute leukemia [1], and CMV seronegative acute leukemia may benefit from CMV seropositive donor for reduction of relapse [2–5]. Another large cohort studies revealed CMV infection showed lower leukemia relapse at early and late post-transplantation periods that were independent of acute or chronic graft-versus-host disease (GVHD) [6–8].

There have been several hypotheses for this phenomenon; First, CMV reactivation itself might have a direct anti-leukemic effect with evidence that CMV can infect CD33-positive hematopoietic progenitor cells [9]. Second, the effect could be mediated by stimulation and expansion of CMV-specific donor T-cells [10–13], but this was not supported by the findings that there were no different outcomes in CMV seropositive patients according to the donor's serological status [7]. In addition, another data showed that adoptive immunotherapy with CMV-specific T-cells showed no graft-versus-leukemia (GVL) effect [14]. Third, anti-leukemic effect might result from subclinical CMV viremia without antiviral therapy which showed a stimulatory effect on natural killer (NK) cells that might enhance GVL effect. It was shown that CMV has strong effects on the NK cell killer-immunoglobulin-like receptors (KIR-receptor) repertoire, and may enhance NK cell activity against tumor cells. In addition, avoiding early antiviral therapy might benefit for maintenance of engraftment and early immune reconstitution [15–18]. Fourth, one of the most likely explanations for the phenomenon, that gamma-delta T-cells elicited by CMV reactivation could cross-recognize both CMV-infected cells and leukemic cells [19].

We tried to evaluate a relationship between CMV reactivation and long-term relapse of acute leukemia in association with preemptive antiviral therapy. We retrospectively analyzed adult acute leukemia patients treated with allogeneic hematopoietic stem cell transplantation (allo-HSCT) in first remission and excluded factors like GVHD and early deaths. Finally, we tried to show treatment outcomes according to CMV reactivation in three different subgroups; undetected group, treated group, and untreated group without regarding the level of CMV reactivation.

## RESULTS

### Baseline characteristics

Patients were classified into three groups – Group 1, untreated or treatment-delayed CMV reactivated group with relatively low RQ-PCR (*n* = 203), Group 2, treated CMV reactivated group (*n* = 140), Group 3, CMV undetected group (*n* = 46). Baseline characteristics of the 3 groups are represented in Table 1. The group treated with antiviral therapy for CMV-reactivation was consisted of significantly older patients compared to untreated group (*p* = 0.061) or CMV-undetected group (*p* = 0.010). The duration from stem cell infusion to CMV reactivation was not significantly different between the treated and untreated CMV-reactivated groups (median 36 *vs*. 31 days). Among the 343 patients with CMV reactivation, most of the patients were identified with CMV reactivation within 100 days (range: 12∼99 days) except 6 patients (range: 102∼172 days). CR rate, duration from the first induction chemotherapy to allo-HSCT, and the proportion of adverse-risk karyotype were not significantly different among the 3 groups. And it was also not different in each acute myeloid leukemia (AML) and acute lymphoid leukemia (ALL) subgroup analysis. We checked the CMV serostatus of the donor and the patients before HSCT, and only 2 patients (0.5%) and 9 donors (2.3%) were negative for CMV IgG antibody.

**Table 1:** Baseline characteristics of the entire acute leukemia patients divided in accordance of the degree of the post-HSCT CMV reactivation and preemptive antiviral treatments

	Delayed or untreated CMV reactivated group (*n* = 203)	Treated CMV reactivated group (*n* = 140)	CMV undetected group (*n* = 46)	*p* (* < 0.05)
Age (median, range)	37.9^1,2^ (18–65)	40.6^2^ (18–65)	35.0^1^ (15–58)	0.024*
Gender (Male (%))	114 (56.2%)	80 (57.1%)	27 (58.7%)	0.947
Time to CMV reactivation (day)	36 (13–170)	31 (12–181)	0.0 (0.0–0.0)	0.091
Maximum CMV titer (median, range)	6,040 (500–25,244)	69,466 (10,330–5,024,065)	0.0 (0.0–0.0)	< 0.001*
AML (*n* = 197)	95 (46.8%)	71 (50.7%)	31 (67.4%)	
CR after 1st CTx	84 (88.4%)	62 (87.3%)	30 (96.8%)	0.335
Time to HSCT (mo)	5.1 (3.7–8.8)	5.4 (3.7–11.5)	4.9 (3.9–7.7)	0.080
Cytogenetic risk				
Favorable (*n* = 44)	21 (22.2%)	12 (16.9%)	11 (35.4%)	0.115
Intermediate (*n* = 125)	60 (63.2%)	47 (66.2%)	18 (58.1%)	0.724
Adverse (*n* = 28)	14 (15.8%)	12 (16.9%)	2 (6.5%)	0.363
ALL (*n* = 192)	108 (53.2%)	69 (49.3%)	15 (32.6%)	
CR after 1st CTx	92 (85.2%)	58 (84.1%)	14 (93.3%)	0.650
Time to HSCT (mo)	5.2 (3.8–10.2)	5.4 (3.5–9.2)	5.1 (3.7–7.8)	0.355
Cytogenetic risk				
Standard (*n* = 71)	39 (36.1%)	25 (36.2%)	7 (46.7%)	0.721
Adverse (*n* = 121)	69 (63.9%)	44 (63.8%)	8 (53.3%)	0.721
CMV serostatus				
Donor CMV IgG(+)	199 (98.0%)	136 (97.1%)	45 (97.8%)	0.864
Patient CMV IgG (+)	203 (100%)	140 (100%)	44 (95.7%)	-
Donor type				
MSD (*n* = 204)	111 (54.7%)	60 (42.9%)	33 (71.7%)	0.002*
URD (*n* = 155)	87 (42.9%)	57 (40.7%)	11 (23.9%)	0.058
FMT (*n* = 23)	4 (2.0%)	18 (12.9%)	1 (2.2%)	< 0.001*
DCBT (*n* = 7)	1 (0.5%)	5 (3.6%)	1 (2.2%)	0.106
HSCT intensity				
MAC (*n* = 261)	149 (73.4%)	80 (57.1%)	32 (69.6%)	0.007*
RIC (*n* = 128)	54 (26.6%)	60 (42.9%)	14 (30.4%)	
HSCT conditioning				
TBI contained (*n* = 312)	161 (79.3%)	111 (79.3%)	40 (87.0%)	0.473
Non-TBI (*n* = 77)	42 (54.5%)	29 (37.7%)	6 (7.8%)	
HSCT Source				
BM (*n* = 185)	96 (47.3%)	60 (42.9%)	29 (63.0%)	0.059
PB (*n* = 197)	106 (52.2%)	75 (53.6%)	16 (34.8%)	0.070
Cord blood (*n* = 7)	1 (0.5%)	5 (3.6%)	1 (2.2%)	0.106
GVHD prophylaxis				
Tacrolimus (*n* = 185)	92 (45.3%)	80 (57.1%)	13 (28.3%)	0.002*
Cyclosporine (*n* = 204)	111 (54.7%)	60 (42.9%)	33 (71.7%)	
ATG (*n* = 110)	49 (24.2%)	51 (36.5%)	10 (21.8%)	0.026*
Non-ATG (*n* = 279)	154 (55.2%)	89 (31.9%)	36 (12.9%)	
Early events < 100 days	30 (14.8%)	17 (12.1%)	4 (8.7%)	0.497
Acute GVHD (*n* = 221)	109 (53.7%)	93 (66.4%)	19 (41.3%)	0.015*
Steroid therapy	63 (57.8%)	78 (83.9%)	5 (26.3%)	< 0.001*
Moderate to severe chronic GVHD (*n* = 149)	82 (40.4%)	54 (38.6%)	13 (28.3%)	0.310

^1,2,3^ Different numbers indicate significant difference between groups based on Tukey's multiple comparison test.

Abbreviation: CMV DNA RQ-PCR, Cytomegalovirus deoxyribonucleic acid real-time quantitative polymerase chain reaction; CR, complete remission; HSCT, hematopoietic stem cell transplantation; MSD, matched sibling donor; URD, unrelated donor; FMT, familial mismatched transplantation; DCBT, double cord blood transplantation; MAC, myeloablative conditioning; RIC, reduced intensity conditioning; TBI, total body irradiation; BM, bone marrow; PB, peripheral blood; GVHD, graft-versus-host disease; ATG, anti thymocyte globulin.

Among the 204 patients who received stem cell from matched sibling donors (MSD), CMV reactivation was not detected in 33 patients (16.2%), and only 1 (14.3%) showed no CMV reactivation among 7 patients who underwent double cord blood transplantation (DCBT). In contrast, 11 out of 155 matched unrelated donor (URD)-HSCT (7.1%) and 1 out of 23 familial mismatched transplantation (FMT) cases (4.3%) showed no CMV reactivation. More CMV reactivated cases were identified in URD-HSCT (92.9%, *p* = 0.009) and FMT (95.6%, *p* = 0.132) compared to MSD-HSCT (83.8%). Likewise, CMV-undetected group included more MSD-HSCT cases (71.7%, *p* = 0.002), while treated CMV reactivated group included more FMT cases (12.9%, *p* < 0.001).

With regard to the HSCT intensity, there were more patients who were treated for CMV reactivation (80 out of 261 (46.8%)) in myeloablative conditioning (MAC) group compared to the reduced intensity conditioning (RIC) group (60 out of 128 (30.6%), *p* = 0.002). In contrast, TBI contained regimen and HSCT source did not show differences. In the case of GVHD prophylaxis, as we selectively used cyclosporine for MSD-HSCT, cyclosporine use also showed more proportion of CMV-undetected patients (33 out of 204 cases (16.2%), *p* = 0.005) while tacrolimus showed more proportion of treated CMV reactivation (80 out of 185 cases (43.2%), *p* = 0.005). ATG use also included more treated CMV reactivation (51 out of 110 cases (46.4%)) than non-ATG group (89 out of 279 cases (31.9%), *p* = 0.007).

### GVHD and CMV reactivation

Among the 221 patients who experienced acute GVHD (aGVHD), 146 (66.1%) were treated with steroid therapy with prednisolone (≥ 0.5 mg/kg) and 202 (91.4%) showed CMV reactivation. Among the 202 patients with CMV reactivation, 93 (46.1%) were treated with preemptive antiviral therapy, but 109 (53.9%) who showed fast improvement of GVHD and CMV RQ-PCR decrement in follow-up samples were observed without preemptive antiviral therapy with reducing immunosuppressive agents. Treated CMV reactivated group included more proportion of steroid therapy (83.9%, *p* < 0.007). Among the entire 343 patients who experienced CMV reactivation, moderate to severe chronic GVHD (cGVHD) occurred in 136 (39.7%) patients, and among the 140 treated patients for CMV reactivation, 54 (38.6%) experienced moderate to severe cGVHD which showed no different results compared to other groups (*p* = 0.136).

### Clinical outcomes according to the CMV reactivation and preemptive antiviral therapy

**Figure 1:**
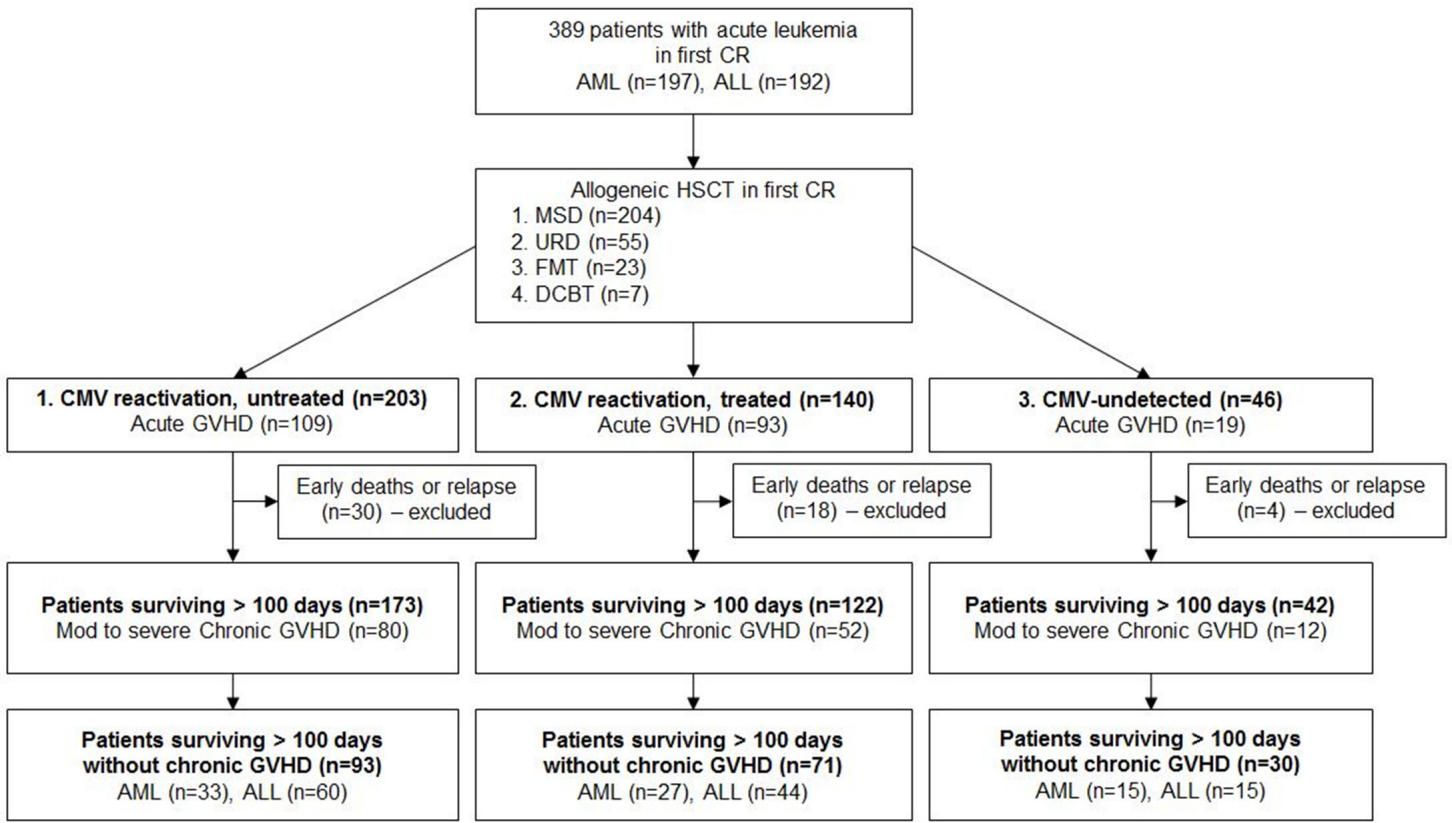
Consort diagram for patient selection Abbreviation: AML, acute myeloid leukemia; ALL, acute lymphoblastic leukemia; CR, complete remission; CMV DNA RQ-PCR, Cytomegalovirus deoxyribonucleic acid real-time quantitative polymerase chain reaction; HSCT, hematopoietic stem cell transplantation; MSD, matched sibling donor; URD, unrelated donor; FMT, familial mismatched transplantation; GVHD, graft-versus-host disease; ATG, anti thymocyte globulin; MMF, mycophenolate mofetil.

Median follow-up duration was 45.0 months (range: 8.5–81.7 months) after stem cell infusion. Although we calculated outcomes in the entire patients initially, and next we excluded patients with early (< 100 days) deaths or relapse because most of the CMV reactivations were identified within 100 days and we tried to identify subsequent clinical outcomes after CMV reactivation (Figure 1). As we mentioned above, large proportion of patients with aGVHD were followed by CMV reactivation, and more steroid treatments were used in treated CMV reactivated group. Early death rate was 8.4% (*n* = 17) in untreated CMV reactivated group, 8.5% (*n* = 12) in treated CMV reactivated group, and 4.4% (*n* = 2) in the CMV-undetected group (*p* = 0.497) and early relapse rates were not different between the 3 groups (6.4% (*n* = 13) vs. 4.3% (*n* = 6) vs. 4.3% (*n* = 2), *p* = 0.657).

**Figure 2:**
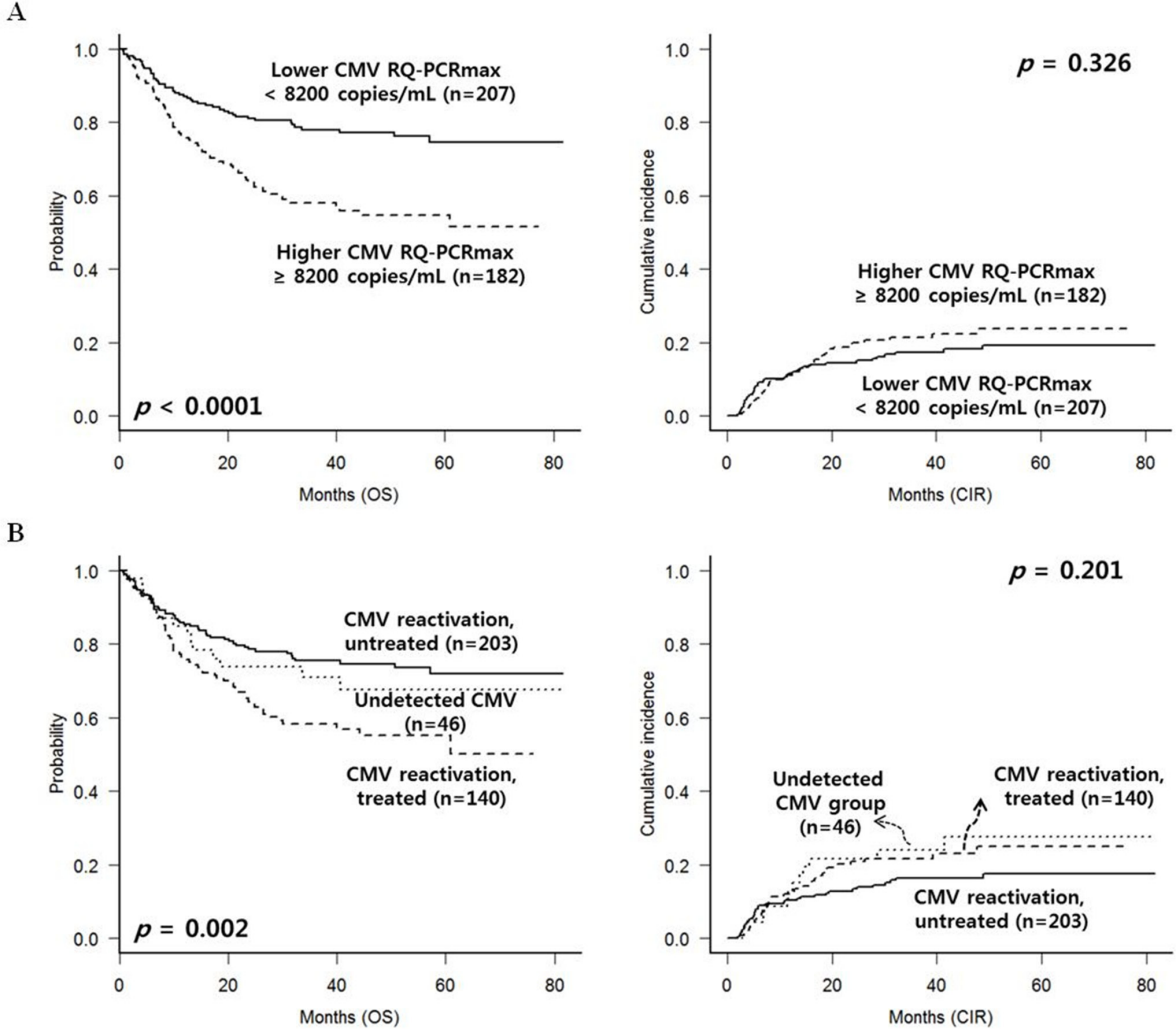
CMV reactivation and treatment outcomes in the entire group (*n* = 389) (**A**) Higher maximal level of CMV RQ-PCR showed adverse effect on OS. However, relapse incidence was not significantly different. (**B**) Among the 3 groups divided based on the CMV RQ-PCR level and preemptive antiviral treatment, treated CMV reactivated group showed the worst OS, while untreated group showed favorable OS with relatively lower relapse rate.

In Figure 2A, in the entire group, CMV reactivation with maximal RQ-PCR level higher than 8200 copies/mL showed significantly poorer overall survival (OS) (*p* < 0.0001) compared to the level below 8200 copies/mL. However, cumulative incidence of relapse (CIR) rate according to the CMV reactivation was not significantly different (*p* = 0.326). The maximal CMV RQ-PCR level higher than 8200 copies/mL was calculated according to the Receive Operating Characteristic (ROC) curve analysis (data not shown). OS and CIR rate of the entire patients divided into 3 groups were calculated in Figure 2B. Treated CMV reactivated group showed the worst 5-year OS (55.1% vs. 67.6% vs. 71.8%, *p* = 0.002). Untreated CMV reactivated group showed relatively lower CIR rate without statistical significance (17.5% vs. 25.0% vs. 27.6%, *p* = 0.201).

**Figure 3:**
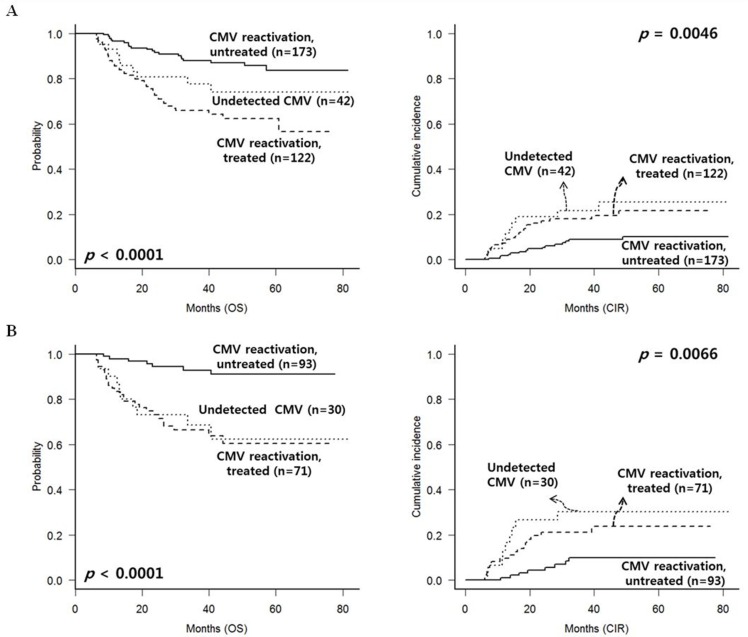
CMV reactivation and treatment outcomes after excluding early deaths or relapse within 100 days (**A**) In the entire group, untreated CMV reactivated group showed favorable OS with lower relapse rate. (**B**) Among patients without significant chronic GVHD, untreated CMV reactivated group showed favorable OS with lower relapse rate.

Next, we analyzed long-term clinical outcomes of patients who have survived without relapse until 100 days post-HSCT (Figure 3A). We identified that untreated CMV reactivated group showed superior 5-year OS (83.8% vs. 74.0% vs. 61.7%, *p* < 0.0001) and lower CIR rate (10.1% vs. 22.1% vs. 25.5%, *p* = 0.0046). Non-relapse mortality rate was higher in treated CMV reactivated group (19.7%, *p* = 0.001) compared to the other two subgroups which showed similar non-relapse mortality rates (6.7% in untreated CMV reactivated group and 4.8% in CMV undetected group). Multivariate analysis was performed in patients excluding early events within 100 days (Table 2). Untreated CMV reactivated group also showed the most favorable OS (HR = 0.39, *p* = 0.019) and event free survival (EFS) (HR = 0.41, *p* = 0.012) with lower CIR rate (HR = 0.27, *p* = 0.002) even compared to CMV-undetected group, and the adverse-risk karyotype showed the worst OS (HR = 2.39, *p* < 0.001) and EFS (HR = 2.37, *p* < 0.001) with higher CIR rate (HR = 2.22, *p* = 0.008).

**Table 2:** Multivariate analysis after exclusion of early deaths or relapse within 100 days (n = 337)

	Multivariate analysis					
	OS		EFS		CIR	
	HR (95% CI)	*p*	HR (95% CI)	*p*	HR (95% CI)	*p*
CMV DNA RQ-PCR						
Undetected group.	1.000	-	1.000	-	1.000	-
Reactivated, untreated	0.39 (0.18–0.86)	0.019*	0.41 (0.20–0.82)	0.012*	0.27 (0.12–0.61)	0.002*
Reactivated, treated	1.44 (0.69–2.97)	0.329	1.32 (0.68–2.59)	0.408	0.85 (0.38–1.89)	0.690
Age (> 40 years old)	1.89 (1.16–3.09)	0.011*	1.70 (1.08–2.66)	0.020*	1.893 (1.04–3.44)	0.036*
HSCT intensity (MAC vs. RIC)	1.84 (0.89–3.82)	0.100	1.66 (0.87–3.18)	0.127	1.85 (0.95–3.61)	0.072
HSCT donor type (MSD vs. others)	1.18 (0.74–1.88)	0.494	0.97 (0.64–1.48)	0.891	0.78 (0.44–1.39)	0.401
Acute GVHD	1.22 (0.63–2.36)	0.543	1.33 (0.74–2.41)	0.337	0.61 (0.34–1.08)	0.089
Chronic GVHD	1.09 (0.68–1.73)	0.727	1.00 (0.65–1.53)	0.997	0.69 (0.38–1.27)	0.237
Adverse-risk karyotype	2.39 (1.49–3.83)	< 0.001*	2.37 (1.54–3.64)	< 0.001*	2.22 (1.24–3.99)	0.008*

* *p* < 0.05

Abbreviation: HR, hazard ratio; CMV DNA RQ-PCR, Cytomegalovirus deoxyribonucleic acid real-time quantitative polymerase chain reaction; HSCT, hematopoietic stem cell transplantation; MAC, myeloablative conditioning; RIC, reduced intensity conditioning; MSD, matched sibling donor; GVHD, graft-versus-host disease.

### Subgroup analysis

In the group of patients surviving after 100 days post-HSCT without cGVHD, untreated CMV reactivated group also showed significantly superior 5-year OS (91.1% vs. 60.1% vs. 62.3%, *p* < 0.0001) and lower CIR rate (9.8% vs. 24.1% vs. 30.2%, *p* = 0.0066), and we identified CMV-undetected group showed significantly higher CIR rate (Figure 3B). In the group of patients surviving after 100 days post-HSCT with moderate to severe cGVHD, CMV-undetected group showed the most favorable OS (all were alive) with only 1 relapse, and treated CMV reactivated group still showed worst OS. However, there were no significant differences in CIR rates between the groups (data not shown).

**Figure 4:**
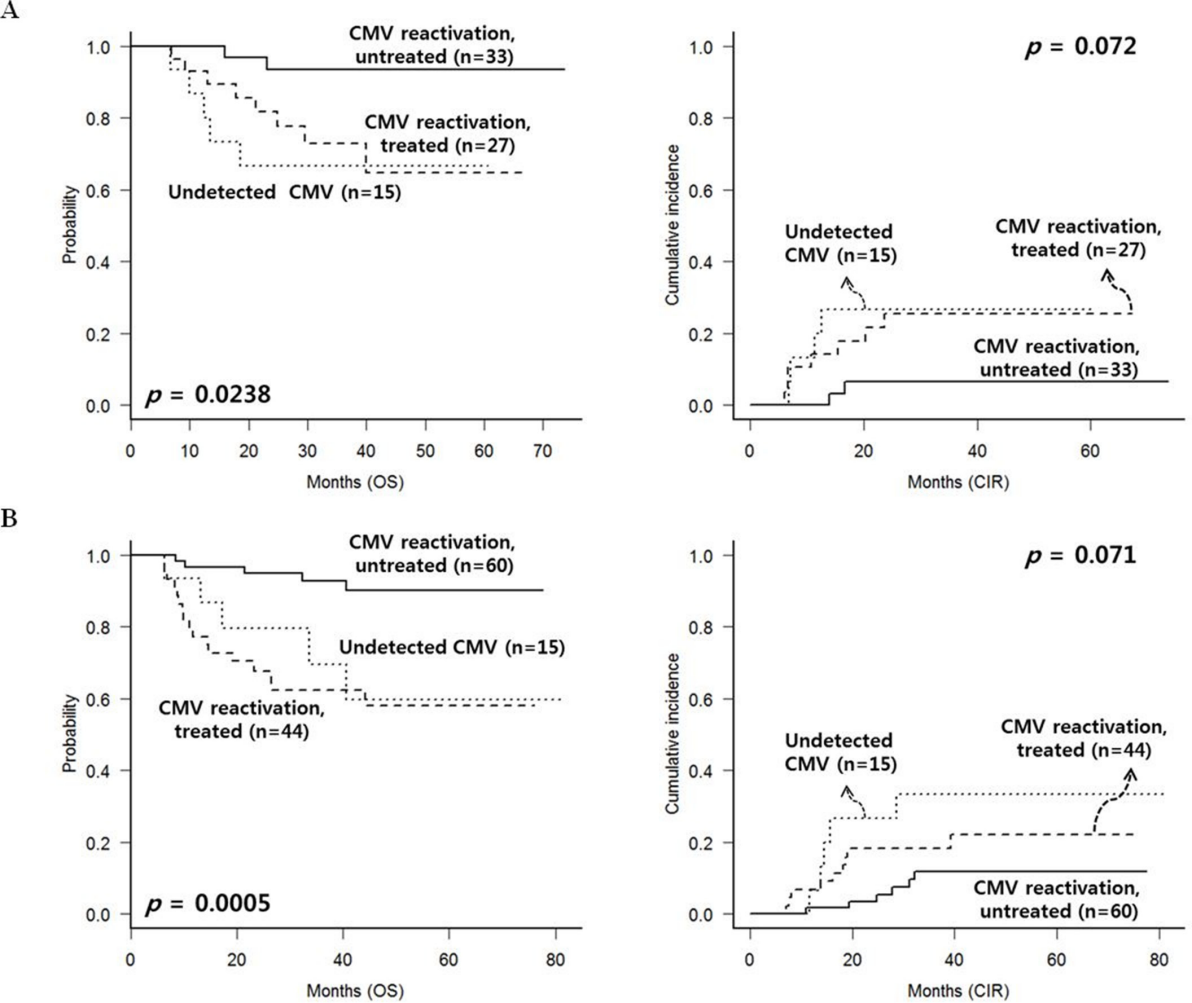
CMV reactivation and treatment outcomes of patients without chronic GVHD, excluding early deaths or relapse within 100 days (**A**) AML subgroup. (**B**) ALL subgroup.

Similarly, we analyzed the outcomes in each subgroup of AML (*n* = 75) and ALL (*n* = 119) after excluding early events and cGVHD. In the AML subgroup (Figure 4A), untreated CMV reactivated group showed the most favorable OS (*p* = 0.024) and lower CIR rate (*p* = 0.072). In the ALL subgroup (Figure 4B), untreated CMV reactivated group also showed favorable OS (*p* < 0.001) with lower CIR rate (*p* = 0.071).

## DISCUSSION

The interaction between CMV infection and leukemia relapse after allo-HSCT has been an area of scientific interest for several years. Our data showed that CMV reactivation may have an additional GVL effect which was identified in the subgroup surviving without relapse after 100 days post-HSCT. Recent data indicated that CMV reactivation within first 100 days after HSCT was associated with a decrease in the risk of early relapse independent of aGVHD in AML, but the result was not statistically significant at 1 year after HSCT. Furthermore, the data also showed that CMV reactivation was not associated with relapse protection at day 100 or 1 year in patients with ALL, lymphoma, CML, or MDS [7]. One study from Korea reported early CMV reactivation concomitant with cGVHD was associated with superior leukemia-free survival [20]. However, the study included only small number of AML patients and the result implicated CMV reactivation showed its favorable effect only in patients with cGVHD.

Unfortunately, most of the previous studies including current analysis were retrospectively designed which may be biased by several affecting factors and included heterogeneous pre-transplant therapies, and variable donors and conditioning regimens. Therefore, we tried to focus on patients with AML and ALL who underwent allo-HSCT in first complete remission (CR1) and excluded cases of early death or relapse within 100 days post-HSCT, and finally adjusted cGVHD to avoid its well-known GVL effect. In addition, our strategy for reducing immunosuppressive agents for all three groups was similar. Post-HSCT immunosuppressive agents were rapidly tapered from 6 weeks after transplantation unless significant GVHD was observed, and the strategy resulted similar incidence of cGVHD in all subgroups. However, as was expected, patients with significant aGVHD showed higher proportion of treated patients due to CMV reactivation. Interestingly, in patients surviving until 100 days post-HSCT without relapse, our data showed untreated CMV reactivated group showed lower relapse rate and better OS compared to treated CMV reactivated group, and even compared to the CMV undetected group.

Preemptive antiviral therapy was performed after considering the patients' infection risk associated with transplantation setting and the severity of GVHD at the time of CMV reactivation. In this specific situation, our data revealed that CMV reactivation without antiviral therapy showed evidence of relapse prevention with possible GVL effects, and it was also proved by multivariate analysis when early death or relapse were excluded. However, in the entire patient group, multivariate analysis identified that young age, cGVHD, and non-adverse-risk karyotype were factors for favorable OS and EFS with lower CIR rate, while the results of untreated CMV reactivated group were not significantly different compared to that of CMV-undetected group. CMV reactivation might not be able to overcome the effect of other significant factors in the whole transplant cohort.

Surveillance of CMV reactivation was mainly performed by pp65 antigenemia assay in previous studies [6, 7, 21]. However, it has been suggested that CMV RQ-PCR has advantages over the antigenemia assay, and we tried to replace the antigenemia assay with RQ-PCR method for CMV monitoring [22]. CMV RQ-PCR method included higher sensitivity, shorter time for procedure, convenient processing for large number of specimens, and the reliable detection is possible during severe neutropenia in early period post-HSCT [23–25]. Although the guidelines for preemptive antiviral therapy base on RQ-PCR method has yet to be established, several randomized studies suggested the cut-off level at 10,000 copies/mL and the use of a DNAemia cut-off avoided unnecessary antiviral therapy without significant CMV disease [25, 26]. We also tried to follow the cut-off for preemptive treatment and ROC curve analysis revealed that maximal CMV RQ-PCR level which significantly affected survival outcome was 8200 copies/mL.

Our data showed possible GVL effect which was proved according to the CMV RQ-PCR level and preemptive antiviral therapy. More sensitive method for CMV detection like RQ-PCR may advance the use of antiviral agent, but the antiviral agent might suppress proliferation and recovery of T-cells [27, 28]. In contrast, delayed initiation or reduction of antiviral agent may have stimulatory effects on NK-cell or T-cell proliferation [15–18, 29–33]. Therefore, if we can maximize anti-leukemic effect, we may propose delaying preemptive antiviral treatment permitting higher CMV RQ-PCR level, despite the strategy may accompany higher risk of fatal CMV disease [34, 35]. It is now required a new prospective study for determining a more reasonable cut-off level for CMV preemptive therapy which may prevent both CMV disease and leukemia relapse along with monitoring CMV specific immune reconstitution, NK cells and several types of T-cell activity.

Among the several hypotheses of anti-leukemic effect of CMV reactivation, up-regulated NK cells in association with CMV reactivation may have an important role. Several reports showed that CMV reactivation has strong effects on the KIR-receptor repertoire, and may enhance NKG2C+ NK cell activity against tumor cells expressing HLA-E [17, 18]. The GVL effect of NK cell is prominent for AML, but ALL is known to be intrinsically resistant to NK recognition [36]. However, our data showed that CMV reactivation without antiviral therapy was also protective for relapse in ALL. This may support a potential role of Vδ2-negative γδT-cells that recognize CMV-infected cells and tumor cells [19]. Besides, they also suggested that not only adoptive transfer of Vδ2-negative γδT-cells but also an application of leukemia reactive Vδ1 TCR–engineered T-cells as alternative therapeutic tools.

Conclusively, our data revealed an anti-leukemic effect of CMV reactivation using RQ-PCR method and suggested a perspective for modification of antiviral treatment strategy in the context of CMV reactivation and leukemia relapse. Particularly for acute leukemia with higher risk of relapse, we suggest delaying preemptive antiviral therapy with reduction of immunosuppressive agents unless the GVHD or CMV disease is expected to be aggravated significantly.

## MATERIALS AND METHODS

### Patients

After approval from the Institutional Review Board of Seoul St. Mary's Hospital (KC13RISI0617), 389 patients with acute leukemia from our database (from 2007 to 2011) were retrospectively analyzed with respect of Declaration of Helsinki. We identified 197 patients with AML and 192 patients with ALL who received allo-HSCT in CR1 after induction, followed by one or two more cycles of consolidation chemotherapy. The median age was 39.0 years (range: 15–65) and there were 221 male patients (56.8%). Cytogenetic risk-stratification was based on NCCN guidelines [37, 38]. For AML, there were 44 (22.3%) patients with favorable-risk karyotype accompanied with *c-kit* mutation or extramedullary manifestations, 125 (63.5%) patients with intermediate-risk karyotype, and 28 (14.2%) patients with adverse-risk karyotype. For ALL, 71 (37.0%) were in the standard-risk and 121 (63.0%) were in the adverse-risk group. All patients underwent allo-HSCT after pre-conditioning based on the protocol set by the Catholic Blood and Marrow Transplantation Center in Korea.

### Treatment details

AML patients were treated with ‘3 + 7’ idarubicin (IDA) plus N^4^-behenoyl-1-β-D-arabinofuranosyl cytosine (BHAC) or cytosine arabinoside (ARA-C) as a remission induction chemotherapy. IDA was administered at a dose of 12 mg/m^2^ and BHAC was administered daily at a dose of 300 mg/m^2^ and the dose of ARA-C was 100 mg/m^2^ continuously infused for 24 hours [39]. ALL patients were treated with hyperfractionated cyclophosphamide (300 mg/m^2^, every 12 hours, days 1 to 3), vincristine (1.4 mg/m^2^, days 4 and 11), IDA (12 mg/m^2^, days 4 and 11), and dexamethasone (40 mg, days 1 to 4 and days 11 to 14), which was mainly based on the hyper-CVAD (cyclophosphamide, vincristine, Adriamycin, and dexamethasone) regimen [40]. Salvage chemotherapy was consisted with ARA-C (2 g/m^2^, every 12 hours, days 1 to 4), mitoxantrone (12 mg/m^2^, days 1 to 4), and etoposide (100 mg/m^2^, days 5 to 7) which was previously reported from our center [41, 42]. In both AML and ALL patients, after achievement of CR, more than one consolidation chemotherapies were administered before allo-HSCT.

Two hundred and four patients (52.4%) received transplants from MSD, and 155 patients (39.8%) received transplants from URD. Twenty three patients with AML received transplants from FMT, and 7 patients with ALL received DCBT. One hundred and eighty-five patients (47.5%) received bone marrow (BM) and 197 (50.6%) received peripheral blood stem cells (PBSC). In AML patients, for RIC regimen, we administered busulfan (6.4 mg/kg) and fludarabine (150 mg/m^2^) with 400 cGy of total body irradiation (TBI) [43]. For MAC regimen, we applied cyclophosphamide (120 mg/kg) combined with 1320 cGy of TBI or busulfan (12.8 mg/kg). For FMT, we administered fludarabine (150 mg/m^2^) and busulfan (6.4 mg/kg) with 800 cGy of TBI and anti-thymocyte globulin (ATG, 5 mg/kg) [44]. For ALL, 1320 cGy of TBI and cyclophosphamide (120 mg/kg) was administered for patients in CR1. Some elderly patients (> 50 years old) were treated with a RIC regimen consisting of fludarabine (180 mg/m^2^) and melphalan (140 mg/m^2^) [41]. For DCBT, we used fludarabine (150 mg/m^2^) and ARA-C (9 g/m^2^) with 1200 cGy of TBI.

GVHD prophylaxis was administered using a calcineurin inhibitor plus a short course of methotrexate (5 mg/m^2^ for tacrolimus and 10 mg/m^2^ for cyclosporine) on D1, D3, D6 and D11. We applied cyclosporine for HSCT from matched sibling donor and tacrolimus for HSCT from unrelated donor, FMT and DCBT. We used acyclovir and itraconazole for prophylaxes, and ciprofloxacin was used for prophylactic gut decontamination. After engraftment, we applied cotrimoxazole for *Pneumocystis jirovecii* pneumonia prophylaxis. For patients without significant GVHD, calcineurin inhibitors were rapidly tapered from 6 weeks post-HSCT and discontinued within 6 months.

### Surveillance of CMV reactivation and preemptive therapy

For surveillance of CMV reactivation, we checked RQ-PCR for CMV DNA after neutrophil engraftment and monitored for CMV reactivation twice a week until discharge. During the follow-up at the outpatient's clinic, patients were monitored weekly or biweekly until the cessation of the immunosuppressive drugs. DNA was extracted from 200 μl of whole blood using QIAamp DNA Blood Mini kit (QIAGEN) and RQ-PCR-based assay for CMV DNA was performed using the LightCycler^®^ 2.0 instrument (Roche Diagnostics, Mannheim, Germany) in accordance with the manufacturer's instructions [22]. The detection limit of the assay is 64.9 copies/mL clinical specimens and the CMV DNA was detected in a linear range from 500 to 1 × 10^7^ copies/mL.

According to the CMV RQ-PCR level, risk-adapted preemptive therapy was conducted to prevent CMV disease. Patients were classified into low- and high-risk group according to both HSCT type and the grade of GVHD based on our previous protocol [22, 45]. High-risk patients were defined as those who had unrelated donors, mismatched related donors, and related donors with aGVHD of grades II–IV or severe cGVHD, and preemptive therapy was considered when DNA copies went over 1,000 copies/mL in two consecutive samples. The remaining patients were classified into low risk, and we started preemptive therapy when DNA copies went over 10000 copies/mL in two consecutive samples. Although RQ-PCR increased over the cut-off, however, some patients without significantly aggravated GVHD were observed with just tapering-off the immunosuppressive agents without applying antiviral agents (i.e. untreated or treatment-delayed CMV reactivated group with relatively low RQ-PCR). Ganciclovir (GCV, 5 mg/kg intravenously every 12 hours) was administered at least for 2 weeks or until CMV RQ-PCR reduced to a level of < 500 copies/mL in 2 consecutive samples. GCV was immediately stopped when the neutrophil count fell to less than 1.0 × 10^9^/L, and replaced by foscarnet (90 mg/kg intravenously every 12 hours). During the CMV preemptive therapy, patients were routinely evaluated for exclusion of CMV retinitis or other diseases and when CMV disease was confirmed, treatment duration was extended to at least 3 weeks or until the resolution of CMV disease.

### Statistical analysis

In this study, we divided patients into three groups according to the CMV RQ-PCR level and whether we treated patients with antiviral therapy in association with GVHD. Between the groups, we compared OS, EFS and CIR rates. All categorical variables were compared using Chi-squared analysis and continuous variables were assessed with the Student's *t*-test and one-way analysis of variance (ANOVA). OS was calculated using Kaplan-Meier analysis, and log-rank analysis was used to evaluate differences between the groups. OS represented the proportion of people who were alive at a specified time from the date of allo-HSCT and EFS took into account death, relapse, loss to follow-up as the result of disease or treatment complications. CIR after HSCT was calculated by cumulative incidence estimation treating non-relapse deaths as competing risks and compared using the Gray test [46]. Survival hazard ratio was calculated using Cox's proportional model. All statistical analyses were performed using SAS 9.2 software (SAS Institute, Inc., Cary, NC) and R software (version 2.15.1, R foundation for statistical Computing, 2012). Statistical significance was determined with *p*-value < 0.05.
